# Transcriptomic Analysis of *Metarhizium anisopliae*-Induced Immune-Related Long Non-Coding RNAs in Polymorphic Worker Castes of *Solenopsis invicta*

**DOI:** 10.3390/ijms241813983

**Published:** 2023-09-12

**Authors:** Junaid Zafar, Hongxin Wu, Yating Xu, Liangjie Lin, Zehong Kang, Jie Zhang, Ruonan Zhang, Yongyue Lu, Fengliang Jin, Xiaoxia Xu

**Affiliations:** National Key Laboratory of Green Pesticide, “Belt and Road” Technology Industry and Innovation Institute for Green and Biological Control of Agricultural Pests, College of Plant Protection, South China Agricultural University, Guangzhou 510642, China; jz_jaam@yahoo.com (J.Z.); scauwhx@stu.scau.edu.cn (H.W.); xuyt1101@stu.scau.edu.cn (Y.X.); liangjie@stu.scau.edu.cn (L.L.); kzh980817@163.com (Z.K.); 13560437669@163.com (J.Z.); sailfin@163.com (R.Z.); luyongyue@scau.edu.cn (Y.L.)

**Keywords:** lncRNA, fire ants, *Metarhizium anisopliae*, biocontrol, host-pathogen interaction, entomopathogenic fungus, miRNAs, *Solenopsis invicta*

## Abstract

Long non-coding RNAs (lncRNAs) represent a class of RNA molecules that do not encode proteins. Generally studied for their regulatory potential in model insects, relatively little is known about their immunoregulatory functions in different castes of eusocial insects, including *Solenopsis invicta*, a notoriously invasive insect pest. In the current study, we used *Metarhizium anisopliae*, an entomopathogenic fungus, to infect the polymorphic worker castes (Major and Minor Workers) and subjected them to RNA sequencing at different intervals (6, 24, and 48 h post-infection (hpi)). Comprehensive bioinformatic analysis identified 5719 (1869 known and 3850 novel) lncRNAs in all libraries. Genomic characteristics analysis showed that *S. invicta* lncRNAs exhibited structural similarities with lncRNAs from other eusocial insects, including lower exon numbers, shorter intron and exon lengths, and a lower expression profile. A comparison of lncRNAs in major and minor worker ants revealed that several lncRNAs were exclusively expressed in one worker caste and remained absent in the other. LncRNAs such as *MSTRG.12029.1*, *XR_005575440.1* (6 h), *MSTRG.16728.1*, *XR_005575440.1* (24 h), *MSTRG.20263.41*, and *MSTRG.11994.5* (48 h) were only present in major worker ants, while lncRNAs such as *MSTRG.8896.1*, *XR_005574239.1* (6 h), *MSTRG.20289.8*, *XR_005575051.1* (24 h), *MSTRG.20289.8*, and *MSTRG.6682.1* (48 h) were only detected in minor workers. Additionally, we performed real-time quantitative PCR and experimentally validated these findings. Functional annotation of *cis*-acting lncRNAs in major worker ants showed that lncRNAs targeted genes such as *serine protease*, *trypsin*, *melanization protease-1*, *spaetzle-3*, etc. In contrast, apoptosis and autophagy-related genes were identified as targets of lncRNAs in minor ants. Lastly, we identified several lncRNAs as precursors of microRNAs (miRNAs), such as miR-8, miR-14, miR-210, miR-6038, etc., indicating a regulatory relationship between lncRNAs, miRNAs, and mRNAs in antifungal immunity. These findings will serve as a genetic resource for lncRNAs in polymorphic eusocial ants and provide a theoretical basis for exploring the function of lncRNAs from a unique and novel perspective.

## 1. Introduction

Innovations in RNA sequencing technology have enabled rapid investigations of protein-coding and non-coding RNA (ncRNA) in vertebrate and invertebrate genomes [1,2,3]. Transcriptome sequencing of various species has revealed that much of the genome undergoes transcription; however, only a fraction of these sequences code for proteins, suggesting that a significant portion embodies ncRNAs [4,5]. NcRNAs are the largest class of RNAs, broadly classified into small ncRNAs (sncRNAs) and long ncRNAs (lncRNAs) [6]. The lncRNAs are transcripts with a length of ≥200 nucleotides (nt). LncRNAs execute their functions through various mechanisms, such as signals, decoys, guides, and scaffolds [7]. They are involved in several biological processes, including epigenetics [8], alternative splicing [9], dosage compensation [10], host-pathogen interactions [11] and gene expression regulation [12]. The discovery of putative lncRNAs in eukaryotes has revealed remarkable variation in expression [13]. In recent years, researchers have identified numerous lncRNA transcripts from several insect species, including *Sogatella furcifera* [14], *Apis mellifera* [15], *Tribolium castaneum* [16], *Bombyx mori* [17] and *Aedes albopictus* [18]. However, most of our functional understanding regarding lncRNAs comes from plants and mammals [19,20] and the lncRNAs in arthropods, mainly eusocial insects, remain largely unexplored.

The red Imported fire ant, *Solenopsis invicta* Buren (Hymenoptera: Formicidae), is an omnivorous soil-dwelling and notoriously invasive ant species [21]. They were inadvertently introduced into the USA early in the 19th century as stowaways’ cargo shipped from their native South American range [22,23]. Since then, they have spread to other parts of the world, including the Caribbean [24], New Zealand [25], Australia [26], Japan [27], South Korea [28] and China [29]. Strong reproductive ability, efficient foraging, aggressive behavior, and adaptability have permitted *S. invicta* to establish and thrive in almost every habitat [30]. The economic impact of fire ant infestations is massive, with the estimated cost of management, medical treatment, and property damages exceeding $6 billion in the US alone [31]. Red imported fire ants are listed among the top 100 invasive species in the world by the International Union for Conservation of Nature (IUCN) and the Invasive Species Specialist Group (ISSG) [32]. To combat the ever-growing threat of fire ants, several integrated pest management (IPM) programs have been devised, mainly centered around the use of synthetic insecticides, though intensive application of pesticides has been detrimental to the environment and the leading cause of resistance development [33,34,35]. These concerns have urged scientists to identify safer, more effective, and eco-friendly biocontrol agents.

*Metarhizium anisopliae*, an insect pathogenic fungus, is an effective biocontrol agent for managing insect pests [36,37,38]. The fungus penetrates the cuticle and proliferates hemolymph, resulting in fungal growth inside the host and ultimately producing and dispersing secondary infectious conidia [39]. Several species of pathogenic fungi have been tested and proven efficacious against red imported fire ants [40,41,42]. Ants are eusocial insects that benefit from social immunity to counter invading pathogens, which is well documented [43,44]. However, in addition to chemical and behavioral defenses, ants also have an innate immune system, which has received limited attention. Insect innate immunity consists of humoral and cellular responses, which involve the production of anti-microbial peptides (AMP), phagocytosis, encapsulation, and other mechanisms [45]. Several molecules, including lncRNAs, play crucial immunoregulatory roles during insect-pathogen crosstalk [46].

Worker ants make up the largest proportion of the colony population. They exhibit caste polymorphism, a dynamic division of labor in which workers are divided into three castes based on their unique physical characteristics. These include Minor (nursing), Medium (foraging), and Major (defense) [47]. Research findings suggest that the age and size of the insects can influence their vulnerability to pathogens [48,49]. In honey bees, immune challenges elicit different responses in nurses and foragers [50]. During aging, *Drosophila melanogaster* shows evidence of upregulation in genes related to pathogen recognition and immune defense [51]. Studies comparing nurses and foragers in *S. invicta* found higher *TpnC* gene expression in foragers, a gene responsible for muscle development, underpinning the relationship between genome and division of labor in ants [52].

However, pathogenic fungi-induced immune responses and the role of lncRNA in governing these responses in different castes of *S. invicta* remain elusive. In the current study, we performed strand-specific RNA-seq of *M. anisopliae*-infected and uninfected Major and Minor workers of *S. invicta* to explore and compare the lncRNA-mediated molecular interactions in different castes. These findings will provide a genetic resource for future functional studies of lncRNAs involved in *S. invicta* immune responses and help develop biotechnology-based targeted control strategies for invasive insect pests.

## 2. Results

To understand the dynamic responses of lncRNAs in *S. invicta* infected with *M. anisopliae*, the Major (D) and Minor (X) worker ants were subjected to RNA-seq analysis at different time intervals (0, 6, 24, and 48 h).

### 2.1. Sequencing Analysis and Quality Assessment

High-throughput RNA-seq generated a total of 2,015,232,916 raw reads from 24 libraries. After stringent quality control (removing low-quality reads, adapters, polyA, and N), we filtered 2,011,446,000 clean reads, which ranged between 99.90 and 99.68% in each library. Clean reads were then aligned to the ribosome database to remove rRNA and subsequently mapped to the *S. invicta* genome, demonstrating a total mapping ratio of up to 94% (Table S1). The mapped reads comprised 66.07–81.39% of coding regions, 17.30–30.26% of introns, and 1.32–3.67% of intergenic regions (Figure S1).

### 2.2. Genomic Characterization and Identification of lncRNAs

**Figure 1:**
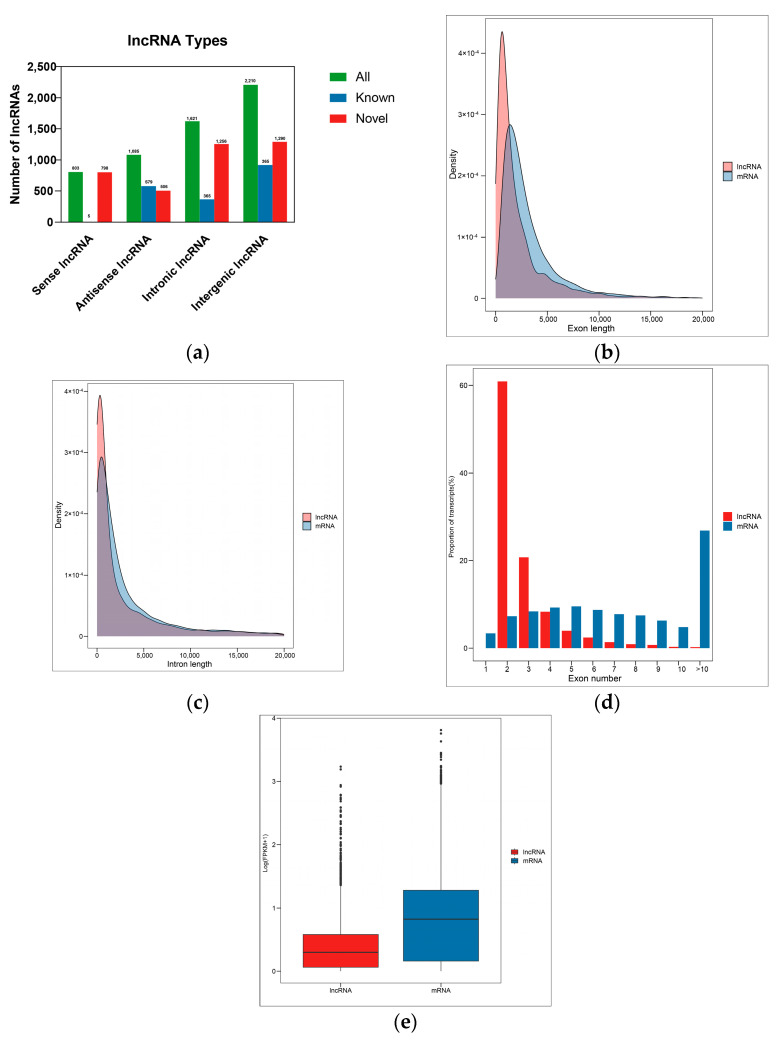
Genomic characteristics of *S. invicta* lncRNAs and structural comparisons with mRNAs (**a**) types of lncRNAs; (**b**) length of exons in lncRNAs and mRNAs; (**c**) length of exons in lncRNAs and mRNAs; (**d**) number of exons in lncRNAs and mRNAs; (**e**) expressional comparisons between lncRNAs and mRNAs.

After stringent filtering (Figure S2), RNA-seq yielded 5719 lncRNAs, including 1869 known and 3850 novel lncRNAs. Based on their relation to the neighboring protein-coding genes, 803, 1085, 1521, and 2210 were classified as sense, antisense, intronic, and intergenic lncRNAs, respectively (Figure 1a). These lncRNAs were shorter in exon and intron lengths than mRNAs and had fewer exon numbers (Figure 1b–d), as documented in other insect species [53,54]. In addition, the expression level of lncRNAs was significantly lower with shorter transcript lengths than mRNAs (Figure 1e). This disparity in expression levels suggests that lncRNAs might function as fine-tuners of gene expression.

### 2.3. M. anisopliae-Responsive lncRNAs in Major and Minor Worker Ants

**Figure 2:**
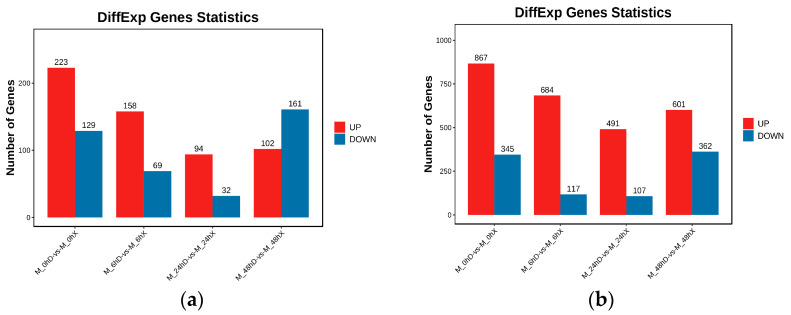
Differential expression patterns of lncRNAs and mRNAs in uninfected and *M. anisopliae*-infected *S. invicta* polymorphic worker ants: (**a**) Number of differentially expressed lncRNAs; (**b**) Differentially expressed mRNAs.

*S. invicta* workers exhibit caste polymorphism and are tasked based on size [55]. To explore distinctions in *M. anisopliae*-induced lncRNAs in different worker castes of *S. invicta*, we identified and compared the expression of lncRNAs in major and minor worker ants. In the uninfected group (M0hD vs. M0hX), 352 lncRNAs (223 upregulated and 129 downregulated) were identified (Figure 2a). Among them, *XR_005574752.1*, *XR_005575300.1*, *XR_005574470.1*, *MSTRG.10559.3*, *MSTRG.20080.1*, *MSTRG.12042.1,* and *XR_005574350.1* were expressed only in major worker ants, while *MSTRG.11994.100*, *MSTRG.4043.16*, *XR_005576277.1*, *MSTRG.18584.2*, *MSTRG.11994.48*, *XR_005576365.1*, *XR_005574765.1*, *MSTRG.3547.1*, *MSTRG.11994.5,* and *MSTRG.10288.1* were only expressed in minor worker ants, implying that even unstressed polymorphic workers have variations in lncRNA abundance and expressions. Though most lncRNAs were shared between both worker castes, their expression varied significantly (Table S2). 

In *M. anisopliae*-infected workers ants, the expression clustering of lncRNAs in M6hD vs. M6hX resulted in 223 differentially expressed lncRNAs (DElncRNAs). Among them, 158 lncRNAs showed upregulation, while 69 lncRNAs were downregulated (Figure 2a). LncRNAs, including *MSTRG.12029.1*, *XR_005575440.1*, and *MSTRG.3547.1*, were abundantly expressed in major worker ants, whereas *MSTRG.11399.1*, *MSTRG.8896.1*, *XR_005574239.1*, and *MSTRG.18864.2* were only present in minor worker ants. Both major and minor worker ants shared the majority of *M. anisopliae*-induced DElncRNAs at 6 hpi, such as *MSTRG.9215.1*, *MSTRG.18880.21*, *MSTRG.13761.5*, *MSTRG.19562.1*, etc. (Table S3), though expressional variations amongst these lncRNAs were evident in both groups. It is worth mentioning that the number of exclusively expressed lncRNAs in major workers remained lower compared to minor workers, possibly due to their strength to resist infections initially. Differential expression profiling at 24 h revealed 126 lncRNAs (94 upregulated and 32 downregulated) (Figure 2a). The comparative analysis demonstrated that lncRNAs *MSTRG.16728.1* and *XR_005575440.1* were only expressed in major worker ants, while *MSTRG.16487.1*, *MSTRG.20289.8*, and *XR_005575051.1* were only present in minor worker ants (Table S4). 

At later stages of infection, M48hD vs. M48hX, 263 DElncRNAs included 102 upregulated and 161 downregulated lncRNAs (Figure 2a). In comparison, *MSTRG.20263.41*, *MSTRG.386.3*, and *MSTRG.11994.5* were among the exclusively expressed lncRNAs in major worker ants, while *MSTRG.12407.1*, *MSTRG.14955.1*, *MSTRG.3646.1*, *MSTRG.20289.8*, and *MSTRG.6682.1* were solely expressed in minor worker ants (Table S5). Interestingly, as the infection progressed (48 hpi), increasing numbers of lncRNAs showed little to no expression in minor workers compared to major workers. Notably, *XR_005575796.1* expression was significantly high in major worker ants, and *MSTRG.4719.1* was abundantly present in minor worker ants at all intervals, suggesting their crucial immunomodulatory functions in response to *M. anisopliae* infection. 

In addition, 1212, 801, 598, and 963 DEmRNAs were identified in M0hD vs. M0hX, M6hD vs. M6hX, M24hD vs. M24hX, and M48hD vs. M48hX, respectively (Figure 2b). The numbers of DElncRNAs and DEGs demonstrated the variation in immune responses among different worker castes in *S. invicta*.

### 2.4. Functional Analysis of M. anisopliae-Responsive lncRNA Target Genes

Recent studies have demonstrated that lncRNAs can regulate gene expression by acting in *cis* on neighboring loci [56]. Here, we investigated the *cis*-regulatory lncRNAs by screening the protein-coding genes as potential targets in the regions located 10 kb upstream/downstream of lncRNAs. GO analyses revealed the putative target genes of lncRNAs in M0hD vs. M0hX, annotated as 24 biological processes-related terms, including cellular process (196), developmental process (110), and signaling (72); 19 cellular components-associated terms, such as cell part (154) and organelle (129); and 10 molecular function-related terms, including binding (156) and catalytic activity (123). The top 20 GO terms are presented in Table S6. In M6hD vs. M6hX, a total of 402 lncRNA-mRNA target pairings were identified, which were annotated as 24 biological processes (i.e., metabolic process, signaling, catalytic activity), 18 cellular components (i.e., cell junction, synapse, extracellular matrix), and 09 molecular functions (i.e., binding, signal transducer activity) associated functions. The top 20 GO terms are presented in Table S7. Similarly, in M24hD vs. M24hX, 231 *cis*-regulatory lncRNA-mRNA target pairings were identified. GO analysis annotation revealed 22 biological processes (i.e., signaling, localization, immune system process), 15 cellular components (i.e., cell part, membrane part), and 08 molecular functions (i.e., catalytic, binding) linked terms. The top 20 GO terms are presented in Table S8. At later stages of infection (M48hD vs. M48hX), 424 *cis*-regulatory lncRNA-mRNA target pairings were identified. GO analyses showed the putative target genes of lncRNAs annotated as 24 biological processes-related terms (i.e., immune system, response to stimulus), 16 cellular components (i.e., synapse, supramolecular fiber), and 09 molecular functions (i.e., signal transducer, structural molecular activity, transporter activity) associated terms. The top 20 GO terms are shown in Table S9. KEGG analysis determined the neighboring genes of lncRNAs in the control group (M0hD vs. M0hX) were enriched in 72 pathways, including mitophagy (05), biosynthesis of amino acids (04), fructose and mannose metabolism (03), starch and sucrose metabolism (02), and dorsoventral axis formation (02). Similarly, 68 pathways were enriched in M6hD vs. M6hX, such as purine metabolism (06), peroxisome (03), Toll and Imd signaling pathways (01), apoptosis (01), and MAPK signaling pathway (01). In M24hD vs. M24hX, ubiquinone and terpenoid-quinone biosynthesis (03), peroxisome (02), and MAPK signaling pathway (01) Additionally, *cis*-regulatory target genes of lncRNAs at M48hD vs. M48hX were enriched in 62 pathways, including the lysosome (06), peroxisome (04), and Hippo signaling pathway (03). The top 20 significantly enriched pathways of all comparison groups are shown in Figure S3. Lastly, we filtered *cis*-acting DElncRNAs that regulated *S. invicta* immune genes in response to *M. anisopliae* infection in both major and minor workers at different intervals. In major worker ants, *Cytochrome P450*, a large family of detoxification enzymes, was consistently regulated by several lncRNAs at all intervals. *XR_005576519.1* was involved in regulating *serine protease* (initiation of the immune response, i.e., melanization); *MSTRG.1438.10* was involved in regulating *Fas-associated factor 1* (FAF1) (immune balance); and *XR_850838.3* was involved in *cis*-regulating *scavenger receptor B1* (SR-B1) (recognition) and *protease-1* (melanization). In minor worker ants, *MSTRG.3312.1* regulated *Cathepsin L* (Gene ID: LOC105208238), a gene crucial to anti-microbial autophagy. *MSTRG.10978.9* was targeting *caspase* (apoptosis), and *MSTRG.12826.9* was *cis*-regulating Jun N-terminal kinase (JNK)-interacting protein 1 (JIP1) (immune pathway). The list of immune-related *cis*-acting DElncRNAs and their target genes is provided in Table 1. Interestingly, we observed that melanization was the key immune response in major worker ants compared to minor workers, whereas apoptosis appeared to play crucial antifungal immune roles.

In addition, *trans*-acting lncRNAs were identified when the absolute correlation was >0.95 between the lncRNAs and mRNAs. Putative target genes of lncRNAs in the uninfected (M0hD vs. M0hX) group were annotated as 24 biological process-related terms, including single-organism process (255), cellular process (250), and biological regulation (180); 18 cellular component-associated terms such as cell (183) and membrane (152); and 11 molecular function-associated terms such as binding (195) and catalytic activity (113). The top 20 GO terms are presented in Table S10. At early stages of infection (M6hD vs. M6hX), the putative targets of lncRNAs were annotated as 24 biological processes (i.e., 196 single-organism processes and 191 cellular processes), 17 cellular components (i.e., 97 membrane parts and 43 macromolecular complexes), and 10 molecular function-associated (i.e., 42 molecular transducer activity) terms. The top 20 GO terms are presented in Table S11. Similarly, in M24hD vs. M24hX, 24 biological processes (i.e., response to stimulus, multicellular organismal process, and signaling), 17 cellular components (i.e., organelle and membrane parts), and 10 molecular functions (i.e., catalytic activity) are related terms. The top 20 GO terms are presented in Table S12. At later stages of infection (M48hD vs. M48hX), the GO analyses identified the target genes of lncRNAs in 24 biological processes (i.e., cellular process, response to stimulus, and localization), 18 cellular components, and 10 molecular function-related terms. The top 20 GO terms are presented in Table S13. KEGG pathway analyses determined that lncRNAs in the uninfected group (M0hD vs. M0hX) were enriched in 45 pathways, including ribosome (18), Wnt signaling pathway (07), Oxidative phosphorylation (05), and glycerolipid metabolism (03). In the early stages of infection (M6hD vs. M6hX), lncRNAs were enriched in 35 pathways such as neuroactive ligand-receptor interaction (16), endocytosis (04), MAPK signaling pathway (02), and Insect hormone biosynthesis (01). In M24hD vs. M24hX, purine metabolism (03), glycerophospholipid metabolism (04), apoptosis (02), and the Hippo signaling pathway (01) In M48hD vs. M48hX, lncRNAs were enriched in 45 pathways, including endocytosis (04), Apoptosis (02), Lysosome (02), and the Hippo signaling pathway (02). The top 20 significantly enriched pathways of all comparison groups are shown in Figure S4.

### 2.5. Detection of S. invicta lncRNAs as Pre-miRNAs

Studies have demonstrated that a significant fraction of lncRNAs can serve as precursors for miRNAs, emphasizing their multifaceted regulatory functions [56,57]. The lncRNAs identified in our studies were subjected to BLAST analysis against miRBase to identify lncRNAs as potential precursors of miRNAs. In total, 21 lncRNAs were identified as possible precursors of 112 miRNAs. A novel lncRNA, *MSTRG.1558.2*, was identified as a precursor of miR-263b, a miRNA widely explored for its involvement in host immunity [58]. LncRNA *XR_005574236.1* was a precursor of two different miRNA families, i.e., miR-210 and miR-6038. Similarly, *XR_005574319.1* was a precursor of miRNAs from multiple families, i.e., miR-100 and miR-7. LncRNA *MSTRG.7696.2* was identified as a precursor of miR-8, a miRNA known for its involvement in the modulation of the Toll pathway and immune homeostasis [59,60]. Moreover, *MSTRG.8464.7*, *MSTRG.10055.1*, *XR_003268631.2*, and *XR_005576483.1* were identified as precursors of miR-bantam, miR-1, miR-10, and miR-14, respectively. These findings presented a complex lncRNA-miRNA-mRNA interaction-based gene regulatory mechanism. A list of miRNAs and their potential precursors is presented in Table S14.

### 2.6. Real-Time Quantitative PCR Analysis

**Figure 3:**
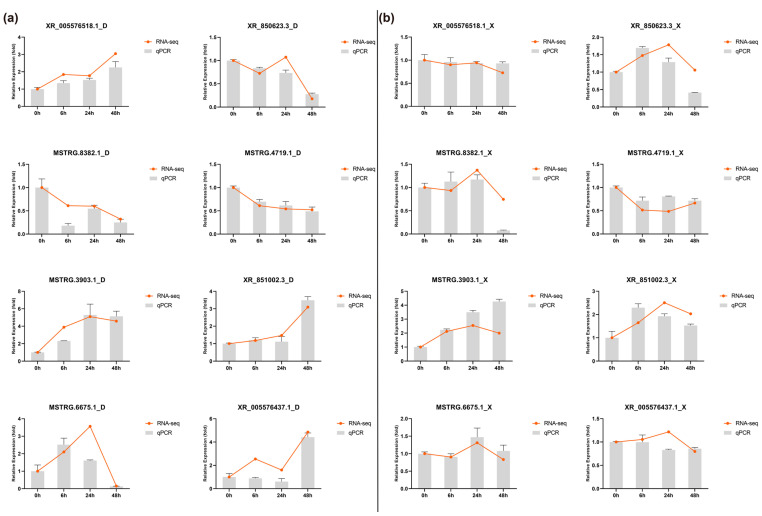
Experimental validation of lncRNAs identified from RNA-seq analysis (**a**) Expression profiling of lncRNAs in major (D) worker ants by RT-qPCR (**b**) Expression profiling of lncRNAs in minor (X) worker ants by RT-qPCR. 0 h represents the uninfected group, while 6, 24, and 48 h depict the expression of lncRNAs in response to *M. anisopliae* infection.

To experimentally validate the identified lncRNAs, RT-qPCR was performed (Figure 3). In total, 08 lncRNAs (*XR_005576518.1*, *XR_850623.3*, *MSTRG.8382.1*, *MSTRG.4719.1*, *MSTRG.3903.1*, *XR_851002.3*, *MSTRG.6675.16*, and *XR_005576437.1*) were randomly selected, and their expression was profiled in both major and minor worker ants. Expressional variations were observed between lncRNAs amongst both worker castes (in both *M. anisopliae*-infected (6, 24, and 48) and uninfected groups (0 h)). For instance, lncRNAs *MSTRG.8382.1* and *XR_005576437.1* exhibited relatively low expression in major worker ants compared to minor workers. Similarly, the lncRNA *XR_850623.3* is highly expressed in major worker ants. These findings demonstrated the expressional variations of lncRNAs even among different castes of eusocial insects. Some discrepancies between RNA-seq and RT-qPCR results were noted, primarily due to the differences between the two techniques.

## 3. Discussion

*S. invicta* is known for its complex social organization and coordinated behaviors. They rely on highly sophisticated communication systems and the division of labor within their colonies to mount a social immune defense against invading pathogens [61]. While the study of immunity in social insects has traditionally focused on colony-wide responses, the individual defenses of its members have received little attention. Polymorphic worker ants comprise the largest proportion of colony populations and play an essential role in colony survival and defense. Since these worker ants are structurally different and have size-based labor divisions, we explored their fundamental molecular differences in immune responses.

Recent research has shed light on the role of lncRNAs in regulating the immune response in insects [46,62]. Here, we employed RNA-seq to profile and compare immune-related lncRNAs in major and minor worker castes of *S. invicta* at different time intervals post-fungal infection. We screened 5719 lncRNAs (1869 known and 3850 novel) from *M. anisopliae*-infected and uninfected *S. invicta* libraries. Analysis of lncRNA genomic features showed similarities with lncRNAs found in other social insects. These included shorter exon and intron lengths, fewer exon numbers, and lower expression levels than protein-coding genes [15,63], demonstrating that these lncRNAs shared common genomic characteristics with other social insect species. However, it is important to note that lncRNAs are often expressed in a spatiotemporal pattern [64]. Therefore, the lncRNAs identified in our study could only be a fraction of the total repertoire, and there is potential to discover plenty more by exploring different stages and pathogens.

Pathogenic stresses can manipulate lncRNA expression [65,66]. Expression profiling showed that several lncRNAs altered their expression at different intervals in response to fungal infection. Similar findings have been reported in other social insects. For instance, *Nosema ceranae*, a microsporidium, altered the expression of several lncRNAs in *A. mellifera* at 7 and 10 days post-infection (dpi) [67]. Our main objective was to identify whether polymorphic worker castes (major and minor) of *S. invicta* have differences in lncRNAs and their expressions under *M. anisopliae* stress. We observed that some lncRNAs are exclusively expressed in major worker castes and vice versa. Evidence suggests that changes in gene expression play a central role in caste differentiation and the phenotype of eusocial insects [68]. Several genes, including immune-related ones, have been reported to exhibit caste-biased expression patterns in social insects [69,70,71]. Transcriptomic analyses performed on a Japanese subterranean termite, *Reticulitermes speratus*, revealed immune response plasticity among all castes [72]. Comparative investigations into two worker subcastes (tiny and large workers) of the leaf-cutting ant *Atta vollenweideri* showed pronounced differences in the expression of immune-related genes [73]. In the long-spined acorn ant (*Temnothorax longispinosus*), a contrasting gene expression pattern was observed between foragers and brood tenders [74]. Caste-specific gene expressions were also observed in two closely related species of fire ants (*S. invicta* and *S. richteri*) [75]. Genomic analysis of two ant species, *Camponotus floridanus* and *Harpegnathos saltator*, revealed caste-, brain, and developmental-stage-specific lncRNAs [63], supporting our findings. Though the caste-specific immunoregulatory potential of lncRNAs is still in its infancy, our results could aid future efforts in understanding the innate immune mechanisms in different castes of eusocial insects, particularly *S. invicta*.

LncRNAs are known to target genes in a *cis* or *trans* manner. The current study identified multiple *cis*-regulatory lncRNAs that target immune-related genes in both worker castes. In major worker ants, immune-related genes, including numerous serine proteases i.e., *melanization protease-1*, *trypsin*, etc., were regulated by several abundantly expressed lncRNAs, implying that cellular immunity, particularly hemolymph melanization, appeared to be the primary immune response. Similarly, the melanization immune response has also been identified in other social insects, e.g., *Reticulitermes flavipes*, against infection by *M. anisopliae* [76]. In contrast, fungus-infected minor worker ants demonstrated that *cis*-acting lncRNAs regulated apoptosis-related genes, including *caspase*, *TRAF*, *JNK-1*, etc. *Cathepsin L*, a gene involved in antimicrobial autophagy [77,78], was also identified as the *cis*-regulatory target of lncRNA in minor worker ants. 

LncRNAs and miRNAs are intricately linked; lncRNAs can serve as precursors for miRNAs and participate in gene regulatory networks [79]. Here, we identified several lncRNAs harboring precursors of miRNAs. Among those, some miRNAs have been functionally studied for their potential involvement in regulating immune-related genes in insects. In *M. anisopliae*-infected *Galleria mellonella*, miR-263b regulates immunity by modulating genes associated with the tumor necrosis factor receptor family [58]. Conserved miR-14, widely explored for its role in *Drosophila* immunity [80], was harbored by lncRNA *XR_005576483.1* in our studies. LncRNAs *XR_005574236.1* and *XR_003268928.2* served as precursors of the miR-6038 and miR-24 families, respectively. These miRNAs are known for their role in neonicotinoid-stressed *A. mellifera* [81]. These multifaceted interactions of lncRNAs with miRNAs and mRNAs demonstrate a complex and multilayered gene regulatory mechanism.

These findings indicate that within eusocial insects, where different castes have specialized roles and distinct physiological characteristics, each caste may exhibit unique individual immune responses, as evidenced by variations in the expression profiles of lncRNAs in our studies. In conclusion, our findings contribute to our understanding of the immune mechanisms governed by lncRNAs in these complex social insect societies and can assist in devising biotechnology-based pest control strategies.

## 4. Materials and Methods

### 4.1. Insects

**Figure 4:**
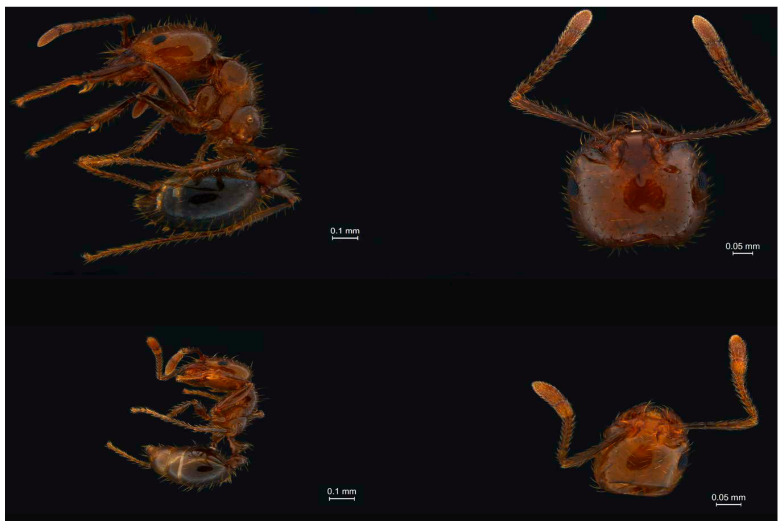
Morphological differences between Major (Top Left = Full body and Top Right = Head) and Minor (Bottom Left = Full body and Bottom Right = Head) worker ants of *S. invicta*.Scale bars are 0.1 mm and 0.05 mm.

The colonies of *S. invicta* were collected from Huangpu District in Guangzhou, Guangdong, China, and brought to the Key Laboratory of Bio-Pesticide Innovation and Application of Guangdong Province, South China Agricultural University, Guangzhou, China. The nests were placed in a 20 L container (internally coated with Fluon^®^ (AGC Chemicals Trading Shanghai, China) to prevent escape). Petri dishes containing minced mealworms, *Tenebrio molitor*, or honey-water (25%) soaked cotton balls were provided as a diet. The colonies were kept under standard laboratory conditions with a 12 h:12 h light:dark photoperiod, approximately 70% relative humidity, and a constant temperature of 26 ± 2 °C. Since worker ants exhibit caste polymorphism, we classified them into two castes “Major” if their head width was >1.000 mm and “Minor” if their head width was <0.595 mm. Head width was measured using a digital micrometer (FB70252; Thermo Fisher Scientific, Waltham, MA, USA) under a stereomicroscope (SMZ-1500; Nikon, Tokyo, Japan) [82]. Figure 4 presents the morphological differences between major and minor workers.

### 4.2. M. anisopliae Infection and RNA Sequencing

Entomopathogenic fungi, *M. anisopliae*, MaqS1902, were kindly provided by Dr. Qiongbo Hu, South China Agricultural University, Guangzhou, China. For the fungal infection, Major (M6hD, M24hD, and M48hD) and Minor (M6hX, M24hX, and M48hX) worker ants were exposed to fungal suspension (5.00 × 10^7^ spores/mL (LC_50_)) by immersion for 4-5 s [83], while the control groups (M0hD/M0hX) were exposed to aqueous 0.05% Tween-80 (Sigma-P1754). The whole-body samples (n = 150/sample) were collected from both groups (Major/Minor) and immediately frozen in liquid nitrogen. TRIzol reagent (Invitrogen, Carlsbad, CA, USA) was used to extract total RNA. The quality of RNA was determined using the RNA Nano 6000 Assay Kit of the Bioanalyzer 2100 system (Agilent Technologies, Palo Alto, CA, USA). A total of 1 μg total RNA per sample was used as input material for the lncRNA library preparation. Strand-specific libraries were generated using the NEBNext^®^ Ultra^TM^ RNA Library Prep Kit for Illumina^®^ (New England Biolabs, MA, USA), following the manufacturer’s recommendations, and index codes were added to attribute sequences to each sample. Briefly, ribosomal RNA (rRNA) was depleted from the total RNA. Fragmentation was carried out using divalent cations at elevated temperatures in the NEBNext First Strand Synthesis Reaction Buffer (5×). The first strand of cDNA was synthesized using a random hexamer primer and M-MuLV Reverse Transcriptase (RNase H-). The second-strand cDNAs were synthesized via DNA polymerase I, RNase H, dNTPs with dUTPs substituted for dTTPs, and buffer. Subsequently, the cDNA fragments (preferentially 250–300 bp) underwent purification using the Apure XP system (Beckman Coulter, Beverly, MA, USA), end-repaired poly(A) addition, and ligation to the Illumina sequencing adapters. To digest the second-strand cDNA, USER^TM^ Enzyme New England Biolabs, MA, USA) was utilized. The digested products were size-selected by agarose gel electrophoresis, PCR amplified, and sequenced using Illumina NovaSeq 6000 by Gene Denovo Biotechnology Co. (Guangzhou, China). 

### 4.3. Read Filters and Transcript Assembly

The raw reads were filtered using fastp (version 0.18.0) to obtain high-quality, clean reads for subsequent assembly and analysis. The filtering process involved the following parameters: (1) removal of reads containing adapters; (2) removal of reads containing more than 10% of unknown nucleotides (N); and (3) removal of low-quality reads containing more than 50% of low-quality bases with a Q-value of ≤20. Bowtie2 (version 2.2.8), a short-read alignment tool, mapped the reads to the rRNA database to remove the remaining rRNAs [84]. High-quality clean reads were mapped to the reference genome of *S. invicta* (GCF_016802725.1) using HISAT2 (version 2.1.0) [85] and transcripts were reconstructed via StringTie (version 1.3.4) [86], which, together with HISAT2, enables the identification of novel genes and splice variants of known genes.

### 4.4. Identification, Annotation and Quantification of lncRNAs

The reconstructed transcripts were aligned to the reference genome and categorized into fifteen groups using Gffcompare. Transcripts with one of the class codes “u, j, i, x, o” were described as novel. The following parameters were employed to identify reliable novel transcripts: a transcript with a length ≥200 bp and an exon number of more than 2. Novel transcripts were then aligned to Nr, Gene Ontology (GO), and Kyoto Encyclopedia of Genes and Genomes (KEGG) to obtain functional protein annotations. Two software tools, Coding-Non-Coding Index (CNCI) and Coding Potential Calculator (CPC2), were utilized to assess the protein-coding potential of the novel transcripts using their default parameters. The intersection of both was chosen as reliable lncRNAs and subsequently classified into intergenic, bidirectional, intronic, antisense, and sense lncRNAs. Different types of lncRNAs may function in various biological processes. The expression level of transcripts was normalized to FPKM (Fragments Per Kilobase per Million mapped fragments), eliminating the influence of transcript length and sequencing depth on the calculation and enabling direct comparison of transcript expression differences among samples.

We conducted a differential expression analysis between the two groups using DESeq2 [87]. Transcripts with a false discovery rate (FDR) < 0.05 and a fold change value ≥ 2, were identified as DElncRNAs.

### 4.5. Prediction of Pre-miRNAs and Target Genes of lncRNAs

LncRNAs can undergo processing by cellular machinery to generate precursor microRNAs (miRNAs), which function as post-transcriptional regulators. To detect potential precursor miRNAs, lncRNAs were aligned to miRBase (version 21) and selected with an identity of > 90%. In addition, miRPara (version 6.3), a software based on the support vector machine (SVM) method, was also used to predict miRNA precursors [88].

LncRNAs exert their gene regulatory functions in either a *cis* or *trans* manner [89]. The *cis*-acting lncRNAs regulate their neighboring genes [90], whereas the *trans*-regulatory lncRNAs function by interacting with co-expressed genes. In the current study, we searched for the protein-coding genes located in regions 10 kb upstream/downstream of the identified lncRNAs to predict their *cis*-regulatory roles. Additionally, we searched for co-expressed genes of lncRNAs for functional analysis. Next, the GO database and KEGG pathway enrichment analyses of all genes were performed for functional annotation. The *p*-value ≤ 0.05 was set as the threshold to determine the significant enrichment of the gene sets.

### 4.6. Real-Time Quantitative PCR Analysis

To validate RNA-sequencing findings, we randomly selected lncRNAs from major and minor worker ants from different groups and profiled their expression by RT-qPCR using a Bio-Rad iQ2 optical system (Bio-Rad, Hercules, CA, USA) and SsoFast EvaGreen Supermix (Bio-Rad, Hercules, CA, USA). In total, 1 μg total RNA was reverse transcribed at 42 °C for 1 h using 1 mL of M-MLV reverse transcriptase (Promega, Madison, WI, USA) according to the manufacturer’s protocol. RPL18 was used as an internal control. The reaction protocol is as follows: 95 °C for 30 s, 40 cycles of 95 °C for 5 s, and 55 °C for 10 s, and a dissociation curve was generated from 65 to 95 °C for 30 s to confirm the purity. Data analysis was performed using the 2^−ΔΔCT^ method. Primers used in this study are listed in Table S15.

## Figures and Tables

**Table 1:** Immune-related cis-acting DElncRNAs and their target genes in polymorphic castes of *S. invicta* at different time intervals.

lncRNA	Target Gene ID	Gene Strand	Target Description	Abundant Expression (Major/Minor Ants)
M6hD vs. M6hX				
*XR_850979.3*	LOC113004832	-	*Cytochrome*	Major
*XR_005575015.1*	LOC105207451	+		
*XR_005575418.1*	LOC105193078	-	*AChE*	Minor
*XR_005576519.1*	LOC113004380	-	*Serine protease*	Major
	LOC120359735	-		
	LOC105196739	-	*CLIP protease 2*	
	LOC105204892	-		
*MSTRG.1438.10*	LOC105201965	+	*FAF1*	Major
*MSTRG.3312.1*	LOC105208238	+	*Cathepsin L*	Minor
*MSTRG.4719.1*	LOC105204661	+	*Cytochrome P450*	Minor
*MSTRG.4919.3*	LOC105204695	+	*AhR*	Major
*MSTRG.6347.1*	LOC105200144	+	*TRAF*	Minor
*MSTRG.8317.1*	LOC105205263	-	*Aminopeptidase N*	Minor
*MSTRG.16558.2*	LOC105206459	+	*Cytochrome P450*	Major
*MSTRG.16671.16*	LOC120359317	+		Major
M24hD vs. M24hX				
*XR_850979.3*	LOC113004832	-	*Cytochrome P450*	Major
*MSTRG.19934.21*	LOC105202834	+		
	LOC105202818	+		
*MSTRG.4719.1*	LOC105204661	+		Minor
*MSTRG.18135.1*	LOC105201710	+		Minor
*XR_005575418.1*	LOC105193078	-	*AChE*	Minor
*MSTRG.10978.9*	LOC105206581	+	*Caspase*	Minor
	LOC105207826	+		
	LOC105194248	+		
M48hD vs. M48hX				
*XR_005574228.1*	LOC105200920	-	*Serine protease*	Major
*XR_005575418.1*	LOC105193078	-	*AChE*	Minor
*MSTRG.12826.9*	LOC105194452	+	*JNK-interacting protein 1*	Minor
*XR_005574580.1*	LOC105204409	-	*Cytochrome P450*	Minor
*MSTRG.8353.3*	LOC105193893	+		Minor
*XR_850979.3*	LOC113004832	-		Major
*MSTRG.4719.1*	LOC105204661	+		Minor
*MSTRG.19934.21*	LOC105202834	+		Major
	LOC105202818	+		
*MSTRG.16671.16*	LOC120359317	+		Minor
*MSTRG.18075.1*	LOC105201710	+	*Cytochrome b5*	Major
		+		Minor
*MSTRG.18135.1*		+		
*XR_850838.3*	LOC105197801	-	*TRAF3*	Major
	LOC105193099	-	*Ankyrin-1*	Major
	LOC105193095	-	*SR-B1*	Major
*XR_005576517.1*	LOC105204787	-	*Melanization protease* *-1*	Major
	LOC113004380	-	*Serine protease*	
	LOC120359735	-		
	LOC105196739	-	*CLIP serine protease-2*	
	LOC105194356	-	*Transmembrane protease serine*	
*XR_005576437.1*	LOC105193099	-	*Ankyrin-1*	Major
*MSTRG.10978.9*	LOC105206581	+	*Caspase-1*	Minor
	LOC105207826	+		
	LOC105194248	+		
*MSTRG.6344.3*	LOC105200144	+	*TRAF*	Minor
*MSTRG.12460.18*	LOC105198898	-	*Trypsin-1*	Major
*MSTRG.12460.53*		-		Major
*MSTRG.12460.67*		-		Major
*MSTRG.14976.1*	LOC113005542	-	*Spaetzle-3*	Major

## Data Availability

The raw reads have been submitted to the Short Read Archive (SRA) database of the National Center for Biotechnology Information (NCBI) under the accession numbers PRJNA981300.

## Supplementary Materials

The following supporting information can be downloaded at: https://www.mdpi.com/article/10.3390/ijms241813983/s1.
