# Supplementary material for: Transcriptomic Analysis of Metarhizium anisopliae-Induced Immune-Related Long Non-Coding RNAs in Polymorphic Worker Castes of Solenopsis invicta
Source: Int J Mol Sci. 2023 Sep 12;24(18):13983. doi: 10.3390/ijms241813983 (PMC10531276; doi:10.3390/ijms241813983)
Supplement: Supplementary file 1 [file ijms-24-13983-s001.zip › Table S10 Top 20 GO categories enriched by trans-regulatory target genes of lncRNAs in M0hD vs. M0hX..pdf]

Table S10. Top 20 GO categories enriched by *trans*-regulatory target genes of lncRNAs in M0hD vs. M0hX.

GO term	Number of enriched genes
Single-organism process	255
Cellular process	250
Binding	195
Cell	183
Cell part	182
Biological regulation	180
Regulation of biological process	170
Metabolic process	157
Membrane	152
Localization	143
Response to stimulus	139
Multicellular organismal process	137
Developmental process	120
Signaling	118
Membrane part	117
Organelle	115
Catalytic activity	113
Cellular component organization or biogenesis	98
Macromolecular complex	68
Organelle part	53

Note: M0hD denotes Major worker ants (uninfected)
M0hX denotes Minor worker ants (uninfected)
