# Supplementary material for: Transcriptomic Analysis of Metarhizium anisopliae-Induced Immune-Related Long Non-Coding RNAs in Polymorphic Worker Castes of Solenopsis invicta
Source: Int J Mol Sci. 2023 Sep 12;24(18):13983. doi: 10.3390/ijms241813983 (PMC10531276; doi:10.3390/ijms241813983)
Supplement: Supplementary file 1 [file ijms-24-13983-s001.zip › Table S11 Top 20 GO categories enriched by trans-regulatory target genes of lncRNAs in M6hD vs. M6hX..pdf]

**Table S11**. Top 20 GO categories enriched by *trans*-regulatory target genes of lncRNAs in M6hD vs. M6hX.

| GO term | Number of enriched genes |
|---|---|
| Single-organism process | 196 |
| Cellular process | 191 |
| Biological regulation | 140 |
| Binding | 139 |
| Regulation of biological process | 132 |
| Cell | 131 |
| Cell part | 130 |
| Membrane | 123 |
| Localization | 114 |
| Response to stimulus | 111 |
| Metabolic process | 111 |
| Multicellular organismal process | 109 |
| Signaling | 100 |
| Membrane part | 97 |
| Developmental process | 90 |
| Catalytic activity | 87 |
| Organelle | 72 |
| Cellular component organization or biogenesis | 66 |
| Macromolecular complex | 43 |
| Molecular transducer activity | 42 |

**Note:** M6hD denotes *M. anisopliae*-infected Major worker ants
M6hX denotes *M. anisopliae*-infected Minor worker ants
