# Supplementary material for: Transcriptomic Analysis of Metarhizium anisopliae-Induced Immune-Related Long Non-Coding RNAs in Polymorphic Worker Castes of Solenopsis invicta
Source: Int J Mol Sci. 2023 Sep 12;24(18):13983. doi: 10.3390/ijms241813983 (PMC10531276; doi:10.3390/ijms241813983)
Supplement: Supplementary file 1 [file ijms-24-13983-s001.zip › Table S12 Top 20 GO categories enriched by trans-regulatory target genes of lncRNAs in M24hD vs. M24hX..pdf]

Table S12. Top 20 GO categories enriched by *trans*-regulatory target genes of lncRNAs in M24hD vs. M24hX.

GO term	Number of enriched genes
Single-organism process	169
Cellular process	163
Biological regulation	119
Binding	118
Regulation of biological process	112
Cell	109
Membrane	108
Cell part	108
Localization	98
Metabolic process	95
Response to stimulus	92
Multicellular organismal process	85
Signaling	84
Membrane part	84
Catalytic activity	75
Developmental process	70
Organelle	57
Cellular component organization or biogenesis	47
Molecular transducer activity	35
Transporter activity	35

Note: M24hD denotes *M. anisopliae*-infected Major worker ants
M24hX denotes *M. anisopliae*-infected Minor worker ants
