# Supplementary material for: Transcriptomic Analysis of Metarhizium anisopliae-Induced Immune-Related Long Non-Coding RNAs in Polymorphic Worker Castes of Solenopsis invicta
Source: Int J Mol Sci. 2023 Sep 12;24(18):13983. doi: 10.3390/ijms241813983 (PMC10531276; doi:10.3390/ijms241813983)
Supplement: Supplementary file 1 [file ijms-24-13983-s001.zip › Table S13 Top 20 GO categories enriched by trans-regulatory target genes of lncRNAs in M48hD vs. M48hX..pdf]

Table S13. Top 20 GO categories enriched by *trans*-regulatory target genes of lncRNAs in M48hD vs. M48hX.

GO term	Number of enriched genes
Single-organism process	223
Cellular process	217
Binding	167
Biological regulation	158
Regulation of biological process	149
Cell part	149
Cell	149
Membrane	136
Metabolic process	133
Response to stimulus	132
Localization	119
Multicellular organismal process	117
Signaling	112
Catalytic activity	106
Membrane part	104
Developmental process	99
Organelle	90
Cellular component organization or biogenesis	75
Macromolecular complex	47
Positive regulation of the biological process	45

Note: M48hD denotes *M. anisopliae*-infected Major worker ants
M48hX denotes *M. anisopliae*-infected Minor worker ants
