# Supplementary material for: Transcriptomic Analysis of Metarhizium anisopliae-Induced Immune-Related Long Non-Coding RNAs in Polymorphic Worker Castes of Solenopsis invicta
Source: Int J Mol Sci. 2023 Sep 12;24(18):13983. doi: 10.3390/ijms241813983 (PMC10531276; doi:10.3390/ijms241813983)
Supplement: Supplementary file 1 [file ijms-24-13983-s001.zip › Table S15 Primers used in this study.pdf]

Table S15. Primers used in the study.

Name	Primer sequences (5´- 3´)
XR_005576518.1_F	AAGGTACCACGCTGTTGTGT
XR_005576518.1_R	AGTCAATGTCGCGCTATTCC
XR_850623.3_F	TCGTGACGTACTTCCTCAAGC
XR_850623.3_R	TGTCCTGCATTCTTCGCACA
MSTRG.8382.1_F	CTGACTGTTGCGGACATCCT
MSTRG.8382.1_R	AACGAGCGCAGCATAGGTTA
MSTRG.4719.1_F	GTTCCTCCGACCTCAACCAG
MSTRG.4719.1_R	ATTATTTTCGTCGCGGTGCG
MSTRG.3903.1_F	TGCCGAGTACAGATTCACTGG
MSTRG.3903.1_R	TGGTCTCATTGTGACTGGAAGA
XR_851002.3_F	TCCACGGTTAACGCTCAGTC
XR_851002.3_R	ACAAGCCGTGGTGTAGCTTT
MSTRG.6675.16_F	AGCAAAACAAACTGTTTAAGGGGA
MSTRG.6675.16_R	AGAGATGATTGGAGTAGGTCTGT
XR_005576437.1_F	CGGCTCATCGCACAACATAG
XR_005576437.1_R	TGCGACTTGTAGCATCGACT
RPL18_F	TTTACGGCTCCTCGTCAAGC
RPL18_R	ACGCAATTCTCCCTTCCAGG
