# Supplementary material for: Transcriptomic Analysis of Metarhizium anisopliae-Induced Immune-Related Long Non-Coding RNAs in Polymorphic Worker Castes of Solenopsis invicta
Source: Int J Mol Sci. 2023 Sep 12;24(18):13983. doi: 10.3390/ijms241813983 (PMC10531276; doi:10.3390/ijms241813983)
Supplement: Supplementary file 1 [file ijms-24-13983-s001.zip › Table S6 Top 20 GO categories enriched by cis-regulatory target genes of lncRNAs in M0hD vs. M0hX..pdf]

Table S6. Top 20 GO categories enriched by *cis*-regulatory target genes of lncRNAs in. M0hD vs. M0hX.

GO term	Number of enriched genes
Single-organism process	199
Cellular process	196
Binding	156
Cell part	154
Cell	154
Metabolic process	150
Organelle	129
Biological regulation	127
Catalytic activity	123
Regulation of biological process	121
Developmental process	110
Multicellular organismal process	96
Response to stimulus	96
Localization	84
Membrane	81
Cellular component organization or biogenesis	80
Signaling	72
Organelle part	68
macromolecular complex	56
membrane part	48

Note: M0hD denotes Major worker ants (uninfected)
M0hX denotes Minor worker ants (uninfected)
