# Supplementary material for: Transcriptomic Analysis of Metarhizium anisopliae-Induced Immune-Related Long Non-Coding RNAs in Polymorphic Worker Castes of Solenopsis invicta
Source: Int J Mol Sci. 2023 Sep 12;24(18):13983. doi: 10.3390/ijms241813983 (PMC10531276; doi:10.3390/ijms241813983)
Supplement: Supplementary file 1 [file ijms-24-13983-s001.zip › Table S7 Top 20 GO categories enriched by cis-regulatory target genes of lncRNAs in M6hD vs. M6hX..pdf]

Table S7. Top 20 GO categories enriched by *cis*-regulatory target genes of lncRNAs in. M6hD vs. M6hX.

GO term	Number of enriched genes
Single-organism process	149
Cellular process	139
Cell part	103
Cell	103
Metabolic process	99
Binding	93
Biological regulation	91
Regulation of biological process	84
Response to stimulus	81
Catalytic activity	80
Organelle	80
Localization	79
Developmental process	68
Multicellular organismal process	63
Membrane	63
Signaling	61
Cellular component organization or biogenesis	51
Membrane part	47
Macromolecular complex	38
Organelle part	35

Note: M6hD denotes *M. anisopliae*-infected Major worker ants
M6hX denotes *M. anisopliae*-infected Minor worker ants
