# Supplementary material for: Transcriptomic Analysis of Metarhizium anisopliae-Induced Immune-Related Long Non-Coding RNAs in Polymorphic Worker Castes of Solenopsis invicta
Source: Int J Mol Sci. 2023 Sep 12;24(18):13983. doi: 10.3390/ijms241813983 (PMC10531276; doi:10.3390/ijms241813983)
Supplement: Supplementary file 1 [file ijms-24-13983-s001.zip › Table S8 Top 20 GO categories enriched by cis-regulatory target genes of lncRNA in M24hD vs. M24hX..pdf]

Table S8. Top 20 GO categories enriched by *cis*-regulatory target genes of lncRNAs in. M24hD vs. M24hX.

GO term	Number of enriched genes
Single-organism process	88
Cellular process	72
Metabolic process	53
Response to stimulus	52
Biological regulation	49
Cell part	49
Cell	49
Catalytic activity	48
Regulation of the biological process	47
Binding	46
Localization	39
Organelle	38
Multicellular organismal process	36
Developmental process	36
Signaling	31
Membrane	31
Cellular component organization or biogenesis	29
Membrane part	22
Positive regulation of the biological process	21
Organelle part	17

Note: M24hD denotes *M. anisopliae*-infected Major worker ants
M24hX denotes *M. anisopliae*-infected Minor worker ants
