# Supplementary material for: Transcriptomic Analysis of Metarhizium anisopliae-Induced Immune-Related Long Non-Coding RNAs in Polymorphic Worker Castes of Solenopsis invicta
Source: Int J Mol Sci. 2023 Sep 12;24(18):13983. doi: 10.3390/ijms241813983 (PMC10531276; doi:10.3390/ijms241813983)
Supplement: Supplementary file 1 [file ijms-24-13983-s001.zip › Table S9 Top 20 GO categories enriched by cis-regulatory target genes of lncRNAs in M48hD vs. M48hX..pdf]

Table S9. Top 20 GO categories enriched by *cis*-regulatory target genes of lncRNAs in M48hD vs. M48hX.

GO term	Number of enriched Genes
Single-organism process	130
Cellular process	124
Binding	94
Metabolic process	92
Cell part	92
Cell	92
Organelle	81
Biological regulation	80
Regulation of biological process	79
Catalytic activity	78
Response to stimulus	76
Localization	66
Developmental process	66
Multicellular organismal process	65
Membrane	55
Cellular component organization or biogenesis	53
Signaling	45
Membrane part	40
Organelle part	37
Positive regulation of the biological process	29

Note: M48hD denotes *M. anisopliae*-infected Major worker ants
M48hX denotes *M. anisopliae*-infected Minor worker ants
