# Supplementary figures and images for: Transcriptomic Analysis of Metarhizium anisopliae-Induced Immune-Related Long Non-Coding RNAs in Polymorphic Worker Castes of Solenopsis invicta
Source: Int J Mol Sci. 2023 Sep 12;24(18):13983. doi: 10.3390/ijms241813983 (PMC10531276; doi:10.3390/ijms241813983)

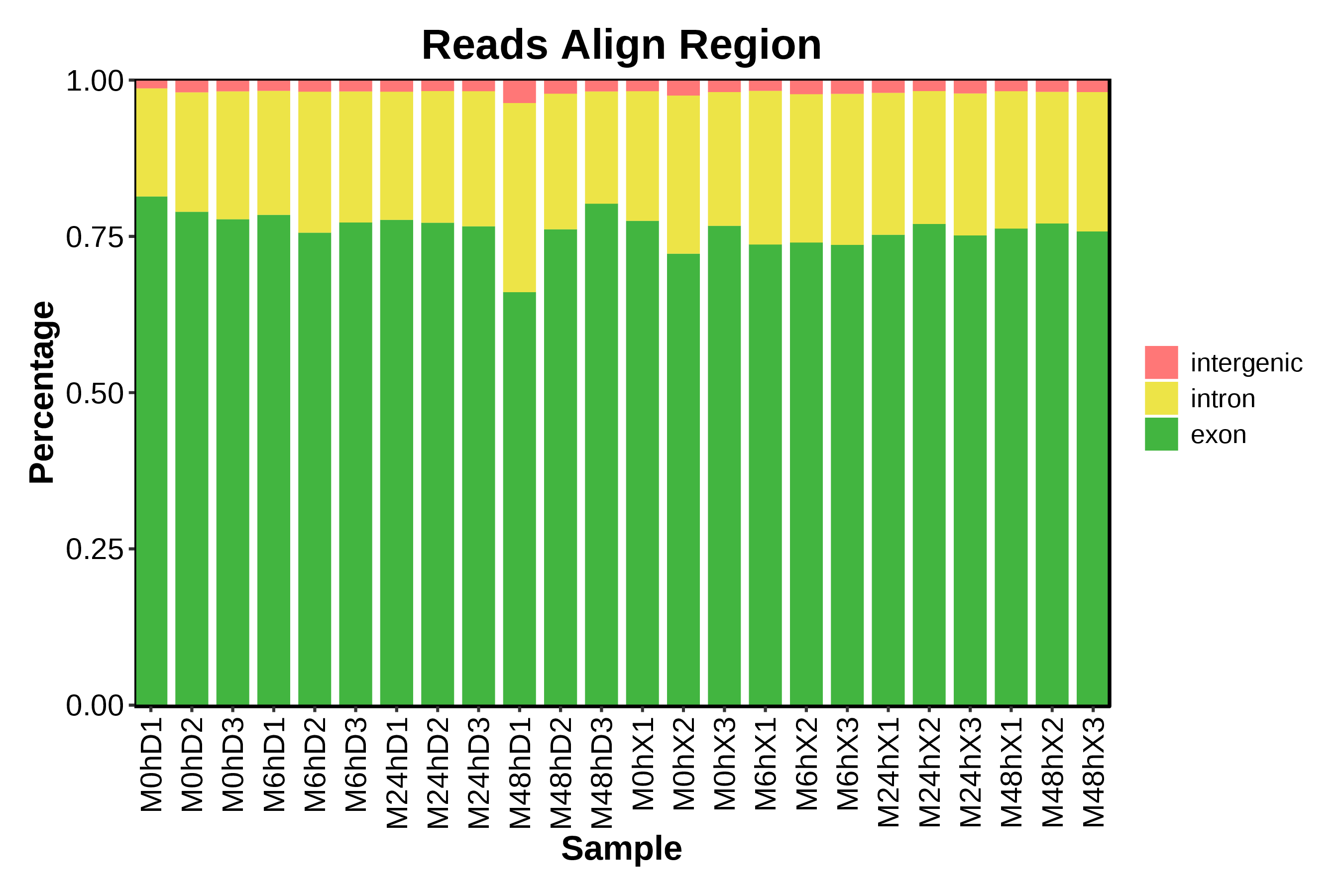

Supplement: Supplementary file 1 [file ijms-24-13983-s001.zip › Figure S1 Percentage of mapped reads onto the regions of intergenic, exons, and introns..png]

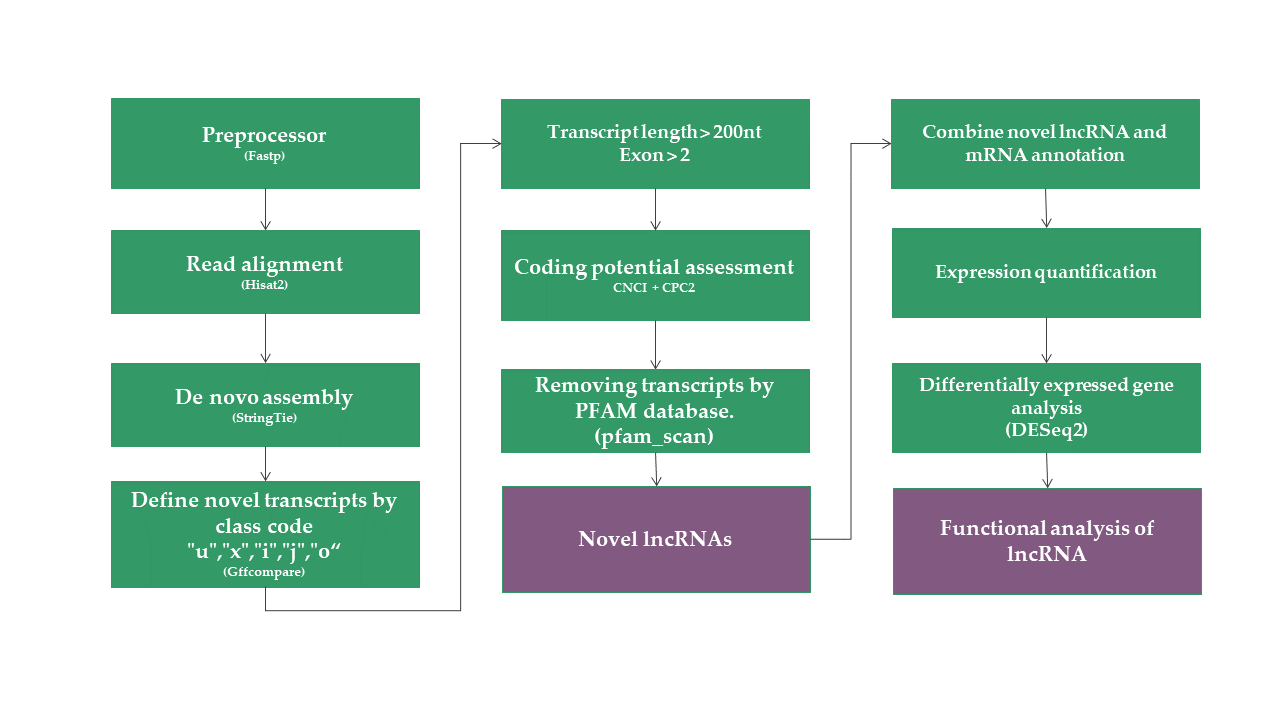

Supplement: Supplementary file 1 [file ijms-24-13983-s001.zip › Figure S2 LncRNA analysis pipeline..png]

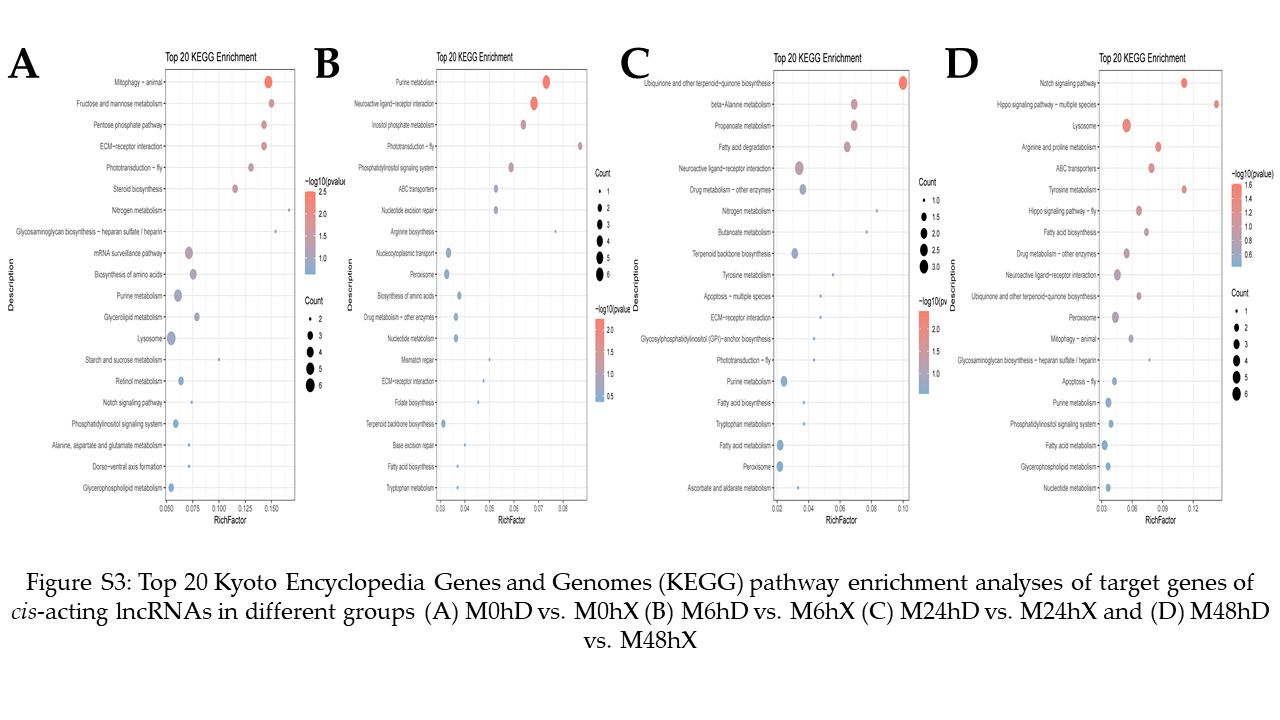

Supplement: Supplementary file 1 [file ijms-24-13983-s001.zip › Figure S3 Top 20 Kyoto Encyclopedia Genes and Genomes (KEGG) pathway enrichment analyses of target genes of cis-acting lncRNAs..PNG]

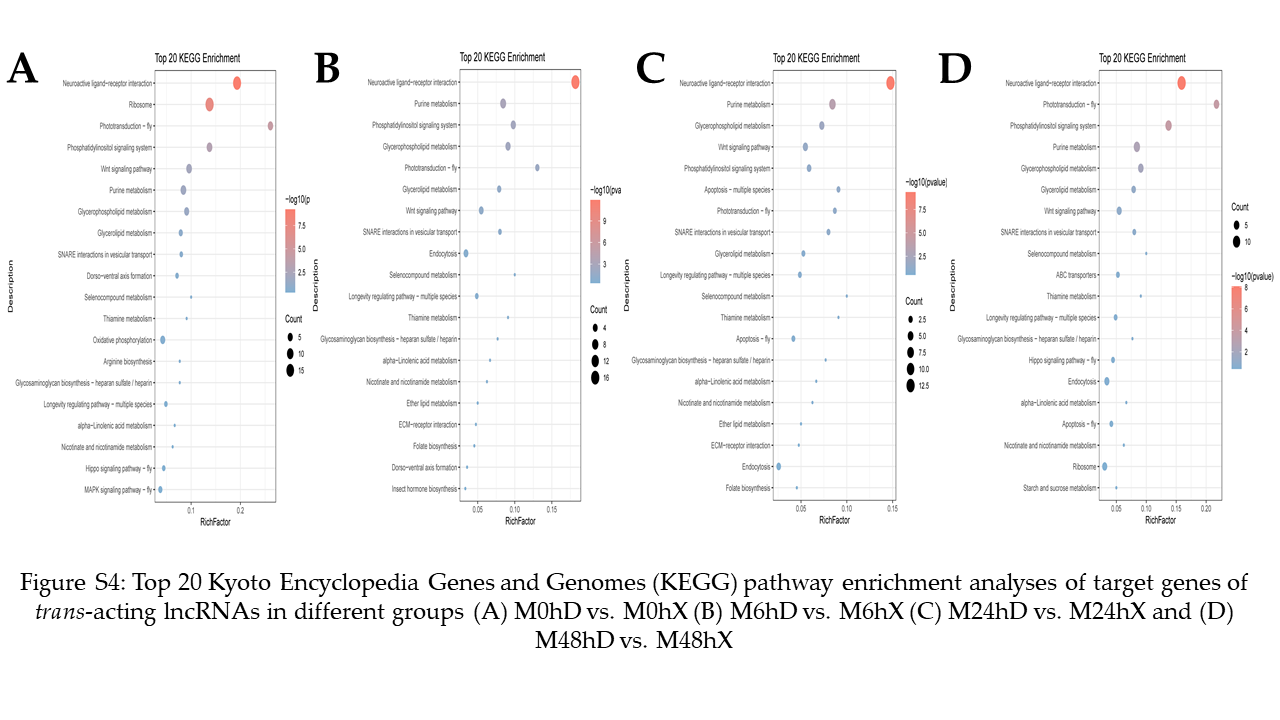

Supplement: Supplementary file 1 [file ijms-24-13983-s001.zip › Figure S4 Top 20 Kyoto Encyclopedia Genes and Genomes (KEGG) pathway enrichment analyses of target genes of trans-acting lncRNAs..PNG]
